# Equivalence of using a desktop virtual reality science simulation at home and in class

**DOI:** 10.1371/journal.pone.0214944

**Published:** 2019-04-11

**Authors:** Guido Makransky, Richard E. Mayer, Nicola Veitch, Michelle Hood, Karl Bang Christensen, Helen Gadegaard

**Affiliations:** 1 Department of Psychology, University of Copenhagen, Copenhagen, Denmark; 2 Psychological and Brain Sciences, University of California, Santa Barbara, CA, United States of America; 3 School of Life Sciences, University of Glasgow, Glasgow, Scotland; 4 School of Applied Psychology, Griffith University, Gold Coast, Australia; 5 Department of Public Health, University of Copenhagen, Copenhagen, Denmark; Indiana University Bloomington, UNITED STATES

## Abstract

The use of virtual laboratories is growing as companies and educational institutions try to expand their reach, cut costs, increase student understanding, and provide more accessible hands on training for future scientists. Many new higher education initiatives outsource lab activities so students now perform them online in a virtual environment rather than in a classroom setting, thereby saving time and money while increasing accessibility. In this paper we explored whether the learning and motivational outcomes of interacting with a desktop virtual reality (VR) science lab simulation on the internet at home are equivalent to interacting with the same simulation in class with teacher supervision. A sample of 112 (76 female) university biology students participated in a between-subjects experimental design, in which participants learned at home or in class from the same virtual laboratory simulation on the topic of microbiology. The home and classroom groups did not differ significantly on post-test learning outcome scores, or on self-report measures of intrinsic motivation or self-efficacy. Furthermore, these conclusions remained after accounting for prior knowledge or goal orientation. In conclusion, the results indicate that virtual simulations are learning activities that students can engage in just as effectively outside of the classroom environment.

## Introduction

The use of simulated labs is booming as companies and educational institutions try to expand their reach, cut costs, increase student understanding, and provide more accessible hands on training for future scientists [1]. Furthermore, there is a strong trend to move educational activities online with the immergence of educational formats such as Massive Open Online Courses (MOOCs) [2,3], providing a number of advantages such as flexibility and the ability to access a large number of students. In 2002 a total of 1,602,970 university students took at least one course online. By 2011 6,714,792 students took one or more online classes, representing an increase 318.9% [4]. This trend is continuously increasing and Arizona State University (ASU) in collaboration with Google Daydream and the educational technology company Labster have recently launched a fully online biology degree course that uses simulations instead of physical lab activities [1]. The course includes 30 three-dimensional lab simulations that are used by students online rather than at the university. Many universities in the US and Europe are following this trend, and as of October 2018, there were 16 universities that were in the process of planning a fully online virtual biology degree with Labster [5].

Reviews and meta-analyses have found that simulations can be effective in promoting knowledge, developing process skills, and facilitating conceptual change and self-efficacy when used as supplements to teaching [6–10]. In addition, students experienced significantly more intellectual intensity, intrinsic motivation, positive affect, and overall engagement when completing game-based homework compared with regular homework [11]. However, little is known about the effect of the context in which simulations are used; in particular, unsupervised home contexts versus supervised classroom contexts. A recent study compared a face-to-face laboratory to a low fidelity virtual laboratory biology simulation in a sample of three hundred undergraduate STEM students over the course of a semester [12]. The results indicated that there were no significant differences on final grades or motivation between the groups. However, students experienced a decline in motivation to learn biology over the course. The study concludes that virtual laboratories may offer an affordable alternative to resource intensive face-to-face laboratories in large-enrollment Biology courses. Another study evaluated the effectiveness of a virtual method of instruction for a general studies, physical science laboratory course [13]. In this study, students were able to choose to either use physical or virtual laboratory exercises. This study also failed to find any differences between the groups on assessment scores, attitudes, or personal preferences. Although these studies suggest that there is equivalence when using simulations across settings, comparisons were made between low fidelity virtual simulations and face-to-face laboratory activities that were not completely identical. Therefore, given the increasing availability of online educational apps, including STEM fields, it is important to explore whether the quality of student learning from these at home is equivalent to engaging with them, under supervision, in the classroom. Thus, the objective of this study was to determine whether the learning and motivational outcomes from learning from high fidelity computer-based science simulation activities during class time are equivalent to doing them at home, self-directed, on the student's own time.

In the present study, we investigate the equivalence of using desktop virtual reality science simulations at home compared to using them under supervision of a teacher in the classroom. The benefits of establishing equivalence is confidence to off-load these activities from the classroom without losing any learning outcome benefits. Furthermore, virtual simulations have the potential of increasing accessibility for all students to high quality laboratory activities [1]. There is increasing interest in extending science simulations from formal (in-school) environments to informal (at-home) environments [14–16], but there is a need to determine whether the context of learning—in school versus at home—affects learning outcomes. In this paper, we explored whether computer-based simulations assigned at home could be as effective for learning and engagement as when they were used during class time under teacher supervision.

### What is the equivalence hypothesis?

The equivalence hypothesis is an extension of Clark's [17] classic theory of learning with media, which holds that instructional methods cause learning regardless of the medium used to deliver the instructional method. Given the growing development of virtual learning environments [18–21], we have extended the concept of instructional medium to include the physical context of learning—that is, learning in virtual reality (VR) at home versus learning in VR at school. The underlying theory is that learning depends on the cognitive activity of the learner during learning, which is primed by the instructional method (e.g., interacting with a science simulation). To the extent that the same cognitive activity is primed both at home and at school, the learning and motivational outcomes should be equivalent. According to the equivalence hypothesis, learning from VR at home should be equivalent to learning from VR at school due to the instructional method (i.e., interacting with the simulation) being the same.

### What is the educational importance of the equivalence hypothesis?

Demonstrating the equivalence of learning outcomes using the same simulation at home or in the classroom effectively allows for more instructional time and flexibility than if all instruction must occur in the classroom. Time is a precious resource in education [22]. Researchers have long recognized the role of instructional time in achieving learning outcomes, as formalized in the time-on-task hypothesis [23]. An important advance in the time-on-task hypothesis is that researchers should focus on engaged learning time—the time the learner is engaged in deep cognitive processing during learning—rather than allocated learning time—the time the teacher provides for learning activities [24]. Spending time at home working on science simulation apps is one way to increase the total amount of engaged time on task. Furthermore, support for the equivalence hypothesis would mean that a larger and more varied sample of students would get access to engaging STEM learning activities.

However, not all at-home activities are equally effective, so we focus on a virtual science lab activity that has been shown to be effective [25–27] and is designed based on cognitive principles of multimedia learning [28]. Virtual laboratory approaches are becoming increasingly popular because they enable delivery of cost-effective, student-centered curricula in the face of increasing student numbers and/or decreased funding [1]; however, there is a paucity of conclusive evidence that these yield equivalent outcomes for learning, motivation, and self-efficacy across different settings [29]. Thus, the educational value of using virtual labs at home depends on the equivalence of these learning and associated outcomes. Even if the equivalence hypothesis holds in general, it is also relevant to investigate if using lab simulations at home vs. the classroom is equivalently effective for different groups of students. Two important variables that could influence learning at home vs. in class are investigated in this study, and are described in more detail below. These include prior knowledge [30], and goal orientation [31].

### Does the equivalence hypothesis hold for students with different levels of prior knowledge?

We investigated the equivalence hypothesis for students with different levels of prior knowledge because several studies have found that educational activities that work for students with low prior knowledge do not necessarily work with high-prior knowledge students [30–35]. The expertise reversal effect refers to the reversal of the effectiveness of instructional techniques for learners with differing levels of prior knowledge [30,35]. For simulations at home to be useful and equivalent to use in a classroom, students with differing levels of prior knowledge need to experience equivalent outcomes.

### Does the equivalence hypothesis hold for students with different forms of goal orientation?

Another individual difference variable that could influence the equivalence hypothesis is goal orientation. Achievement goal orientation refers to the reason why students engage in learning tasks [31]. Achievement goal orientation affects the extent to which students view challenges as opportunities, persist in the face of difficulties, exert effort, are intrinsically motivated to learn, and believe that their abilities can be developed [31]. Holding a mastery orientation, one in which the goal is to learn or master the content or task, is associated with adaptive patterns of learning including higher task efficacy, motivation, effort, persistence, and performance. Performance-approach orientation, where the goal is to demonstrate ability or competence, has also been associated with high performance; however, performance-avoid orientation, where the goal is to avoid demonstrating low ability (i.e., to avoid failing), is associated with poorer performance [36]. Previous research on homework has found that students with high mastery goals did more science and general homework [37]. Those with high performance-avoid goals had higher homework anxiety, which is likely to lead them to avoid engaging in homework [37]. When learning via technology, as in the current study, high mastery goals were associated with less procrastination [38], but there was no difference in the time engaged in the learning compared to those with high performance goals [39]. Given differences in learning engagement and outcomes, especially for homework and when learning online, we examined the equivalence of science simulations at home and in school for students with different levels of mastery, performance-approach, and performance-avoid goal orientation.

## Materials and methods

This research was approved by The University of Glasgow’s College of Medical, Veterinary and Life Sciences Ethics Committee.

### Predictions and analyses

If the equivalence hypothesis is correct we expect that students who engage in computer-based science simulations at home (home group) will have the same outcomes as students who engage in the same simulations in their classroom (classroom group) as measured by tests of learning outcome and motivation (intrinsic motivation and self-efficacy). We report mean differences in learning outcome, intrinsic motivation, and self-efficacy as effect sizes with corresponding 95% confidence intervals. Furthermore, we report the 95% Highest Density Interval (HDI) with respect to the hierarchical t-test model and standardized Bayesian priors [40]. The HDI is a Bayesian method that estimates the difference in means between two groups and provides a probability distribution over the difference.

Furthermore, if the equivalence hypothesis holds, the same effect should be observed for students with different prior knowledge, mastery goal orientation, performance-approach goal orientation, and performance-avoidance. In this study, we predicted that the students who use the virtual simulation at home would not differ from students who take the virtual simulation in the classroom on learning outcome, intrinsic motivation, and self-efficacy after accounting for prior knowledge, and goal orientations (mastery, performance-approach, and performance-avoid). This was addressed using ANCOVA’s adjusting for prior knowledge, and learning goal orientations respectively and by testing interaction effects. We report effect sizes using Cohen's d.

### Participants and design

The sample consisted of 112 students (36 males and 76 females) aged from 18 to 41 (*M* = 20.7 years, *SD =* 4.4), who were part of a biology course at the University of Glasgow. The experiment employed a between-subjects design, in which students in the course were randomly assigned to use the same virtual laboratory simulation on the topic of microbiology at home on their own time (home group; N = 62) or in an assigned classroom setting with teacher supervision (classroom group; N = 50). Only participants who filled out an informed consent to use their data in the study, which is why there was an uneven number of students in the two groups.

### Materials

The materials included a Bacterial isolation virtual lab simulation, participant questionnaire, learning outcome test, and self-report survey (see Appendix 1 for a list of the items used in the study). Unless otherwise indicated all surveys used a 5-point Likert scale ranging from (1) *completely disagree* to (5) *completely agree* and were delivered online using Survey Monkey.

#### Virtual lab simulation

The virtual simulation used in this experiment was entitled ‘Bacterial Isolation’ and was developed and produced by an education technology development firm, Labster (for a short video of the simulation see [41]). This is one lab from a catalogue that is commercially available from the company (www.Labster.com). The simulation is an immersive and interactive digital environment designed to facilitate learning of key concepts and techniques in microbiology at a university or college level. Specifically, the concepts covered were: Understanding the importance of bacterial growth for the investigation of pathological microorganisms, appreciating the need to work under aseptic conditions, understanding the concept of a single colony and why plate-streaking techniques work and understanding the function and role of selective and differential culture media in bacteriology. The key skills learnt were: inoculation of bacterial culture media, colony screening, use of sterile technique and plate streaking for the isolation of single colonies. The simulation allowed the user to work through the procedures in a virtual lab by using and interacting with the relevant lab equipment and the essential content is taught through an inquiry-based learning approach. Students were guided through simulation by a pedagogical agent who gave specific instructions to help students progress smoothly through the simulation [42]. In the simulation the virtual lesson starts off with the learner being presented with a brief introduction to the story behind the lab scenario and the student’s task in resolving the experimental investigation is outlined. The student is tasked with investigating an outbreak of bacterial food poisoning, and has to isolate an antibiotic-resistant strain of the bacterium from chicken litter samples collected from a poultry farm, the suspected source of infection. After being introduced to the principles of selective and differential culture media in microbiology, students are given repeated opportunities to practice streaking out bacteria onto agar plates, incubate them appropriately, and culture isolated colonies free from contamination. This is a key technique in microbiology lab practice and has previously been shown to allow students to learn these skills equally as they would from a real-life lab experience with a teacher [26].

The bacterial isolation simulation includes five different forms of interactivity that are commonly used in multimedia learning environments including: dialoguing, controlling, manipulating, searching, and navigating [43]. In the bacterial isolation simulation dialoguing is achieved through an interaction with the online virtual laboratory instructor and through the optional selection of additional information through wikilinks. Students are also able to control the pace of the bacterial isolation simulation by deciding when to proceed with the experiments, by choosing whether to do further reading when prompted to answer multiple-choice questions, and by controlling the number of times they practice particular components of the lab. Students are required to manipulate the material by having to practice streaking out bacteria on agar plates. This includes selecting the appropriate culture medium, inocula, and controls used in the experiment. The student has to find the correct tools and prepare them adequately to work with the required sterile technique that the protocol demands, and finally has to determine the parameters for successful incubation and growth of the bacteria. Key to the simulation is the student’s ability to use a sterile nichrome loop to streak out bacteria onto an agar plate to be able to grow isolated bacterial colonies, free from contamination. The virtual lab also necessitates the student’s choice for safe disposal of contaminated lab materials. Furthermore it gives the opportunity to engage in information seeking, by providing wikilinks (‘Theory’) to written material giving background to the concepts, techniques and materials they are engaging with. This included expansion on topics of selective and differential culture mediums, clonal growth of bacteria, and resistance and sensitivity of bacteria to antibiotics. Finally, students are also provided with interaction by navigation because the learner is in a virtual lab where they are able to determine the content of the learning episodes by selecting a piece of equipment from various available sources and deciding what to do next by navigating around the virtual lab. The simulation typically takes approximately one hour to complete. Students engaged with the simulation at a point in their course prior to the commencement of a block of ‘real’ labs in which they would have weekly lab practice in the traditional manner. The majority of students would have had no prior experience of these techniques (this data not collected but assumed from knowledge of curriculum) and the theory had not been previously covered in lectures.

#### Pre-test survey

This included demographic questions such as gender and year of study, and scales to measure goal orientation and prior knowledge. Goal orientation was assessed with three scales from the Patterns of Adaptive Learning Scales (PALS [44]). The scales had good reliability with Cronbach’s alphas of .76 for mastery, .86 for performance-approach, and .86 for performance-avoid. Prior knowledge was measured with seven items with response options ranging from one to five intended to assess the students’ existing knowledge of microbiology. The prior knowledge is reported as the average score and the scale had a Cronbach’s alpha of .82.

#### Learning outcome tests

The learning outcome test consisted of 10 multiple-choice questions designed to assess retention of essential material presented in the simulation, and 10 multiple-choice transfer questions designed to assess the participants’ ability to apply what they had learned to new situations. Students received one point for each correct answer and 0 points for selecting an incorrect answer in the retention and transfer tests. The quality of the test was assessed by testing the fit of the items to the Rasch model within the framework of item response theory [45] using RUMM2030 [46]. The results showed that four items did not fit the model. The remaining 16 items showed acceptable fit to the Rasch model (χ2[32] = 45.76, *p* = .055). Furthermore, no multidimensionality was found according to the standard test proposed by Smith [47] so a single total score (out of 16) was used. The test had acceptable discrimination for the sample with a person fit index of .62 and Cronbach’s alpha of .72.

#### Post-test self-report measure

Participants rated their level of intrinsic motivation and self-efficacy in biology. The intrinsic motivation measure was a 5-item scale adapted from Deci and Ryan [48]. The self-efficacy scale was an 8-item scale adapted from the Motivated Strategies for Learning Questionnaire (MSLQ [49]). The scales yielded good reliability, with Cronbach’s alphas of .89 for intrinsic motivation and .92 for self-efficacy.

### Procedure

Students in 2^nd^ and 3^rd^ year microbiology courses (of a 4-year degree program) were invited to participate in the study. The simulation was used to achieve specific learning outcomes for the courses, and as such, all students were expected to engage with it. Students were informed about the study through communications made to them directly during face-to-face lecture time, and via written information on the campus virtual learning environment. Informed consent was taken in accordance with the stipulations required by the university’s College of Medical, Veterinary and Life Sciences Ethics Committee.

Students were randomly assigned to the classroom group or home group, with each group designated to engage with the simulation either at school or at home, respectively. All participants completed the pre-test survey, not more than a week in advance of engaging with the simulation. The classroom group had the simulation scheduled in their timetable, allowing them one hour in a computer lab on campus. The computer lab had the capacity to seat up to 40 students at a time, and the same teaching assistant was present to attend in each session. The teaching assistant was a final year PhD student with extensive lab tutoring experience with these students, and was known to them as an experienced lab scientist in this field (i.e., microbiology). Students in this group could continue to work on the simulation after the hour if they desired. On average, they spent 59.21 minutes on the simulation (range 18 to 127 minutes). The home group was given free choice with regards to where and when they would engage with the simulation outside of school. A total of 52 reported taking the simulation at home and 10 reported taking the simulation somewhere else such as the library, café etc. There were no differences within this group so the group was combined in the remaining analyses. They were able to contact staff if they had operational problems (by registering with the web-site for the simulation) but otherwise were asked to work completely independently of teaching support. Students in this group spent an average of 61 minutes using the simulation (range: 18 to 125 minutes).

On completion of the simulation, students from both groups were asked to complete the post-test, which was emailed to them approximately one week after taking the simulation. Students in the classroom group responded to the post-test an average of 11.37 days after interacting with the simulation, and those in the home group responded an average of 10.73 days after interacting with the simulation.

### Do the groups differ on basic characteristics?

As a preliminary step, we examined whether the home and classroom groups differed on basic characteristics, in spite of random assignment. The average prior knowledge score for the home group (Mean = 3.85, SD = 0.60) and the computer group (Mean = 3.71, SD = 0.70) did not differ significantly with an effect size of 0.14 (95% CI -0.11 to 0.38). Regarding gender, the proportion of men in the home group (19 men, 31%) did not differ significantly from the proportion in the classroom group (17 men, 34%). The difference was 3% (95% CI -14% to 21%).

## Results

### Do the groups differ on learning outcome?

Table 1 shows the summary statistics for the home and classroom groups on the learning outcome test. As can be seen, the home group (Mean = 11.57, SD = 3.14) and the classroom group (Mean = 11.12, SD = 3.03) had an effect size difference of 0.14 (95% CI -0.38 to 0.11), thus, the groups did not differ significantly with respect to learning outcome. The 95% HDI of the difference ranged from -0.78 to 1.60, meaning that 95% of the posterior distribution was in this range. These findings support the equivalence hypothesis and constitute the major empirical contribution of this study.

**Table 1:** Means, standard deviations, minimum, and maximum scores, effect sizes (with confidence intervals, and 95% High Density Interval (HDI) for the dependent variables used in the study.

	Home		Classroom			
	Mean (SD)	Min.-Max.	Mean (SD)	Min.-Max.	Effect size (95% CI)	95% HDI*
Learning outcome	11.57 (3.14)	5–16	11.12 (3.03)	2–16	0.14 (-0.24 to 0.53)	(-0.78 to 1.60)
Motivation	4.10 (0.70)	2–5	4.11 (0.65)	2–5	0.02 (-0.36 to 0.39)	(-0.24 to 0.26)
Self-efficacy	3.70 (0.62)	2–5	3.65 (0.64)	1.38–4.88	0.09 (-0.29 to 0.46)	(-0.21 to 0.19)

*: HDI: Highest Density Interval

### Do the groups differ on self-reported intrinsic motivation and self-efficacy?

Table 1 also shows the summary statistics for the two groups on intrinsic motivation and self-efficacy. For motivation, the home group (Mean = 4.10, SD = 0.70) and the classroom group (Mean = 4.11, SD = 0.65) had an effect size difference of 0.02 (95% CI -0.36 to 0.39). Thus, the groups did not differ significantly with respect to level of intrinsic motivation. The 95% HDI of the difference ranged from -0.24 to 0.26. For self-efficacy, the home group (Mean = 3.70, SD = 0.62) and the classroom group (Mean = 3.65, SD = 0.64) had an effect size difference of 0.09 for self-efficacy (95% CI -0.36 to 0.39). This also indicates that the groups did not differ significantly with respect to level of self-efficacy. The 95% HDI of the difference ranged from -0.21 to 0.19. These findings support the equivalence hypothesis and constitute further empirical contributions of this study.

### Is the equivalence between groups consistent after accounting for prior knowledge?

As can be seen in Table 2, the inclusion of prior knowledge as a co-variate did not lead to differences between the home and the classroom groups on the three dependent measures of learning outcome, intrinsic motivation, or self-efficacy and there was no statistically significant evidence of interactions. More specifically the effect size difference with prior knowledge as a covariate was 0.11 (95% CI -0.28 to 0.50) for the learning outcome (interaction *p* = 0.1999); 0.10 (95% CI -0.28 to 0.48) for intrinsic motivation (interaction *p* = 0.0892); and 0.01 (95% CI -0.37 to 0.39) for self-efficacy (interaction *p* = 0.6397). We conclude that the lack of differences between the home and the classroom groups on learning outcome, intrinsic motivation, and self-efficacy remained after accounting for prior knowledge.

**Table 2:** Effect sizes (with confidence intervals) for the dependent variables without any adjustments, after adjusting for prior knowledge, and after adjusting for goal orientation respectively.

	Effect size (95% CI)				
	Orig.	Adj. for prior knowledge	Adj. for mastery orientation	Adj. for performance-approach	Adj. for performance-avoid
Learning outcome	0.14 (-0.24 to 0.53)	0.11 (-0.28 to 0.50)	0.11 (-0.28 to 0.50)	0.15 (-0.24 to 0.54)	0.16 (-0.23 to 0.54)
Motivation	0.02 (-0.36 to 0.39)	0.10 (-0.28 to 0.48)	0.11 (-0.28 to 0.49)	0.02 (-0.39 to 0.43)	0.00 (-0.40 to 0.40)
Self-efficacy	0.09 (-0.29 to 0.46)	0.01 (-0.37 to 0.39)	Interaction	0.12 (-0.28 to 0.53)	0.11 (-0.30 to 0.52)

### Is the equivalence between groups consistent across learners with different kinds of goal orientations?

**Fig 1:**
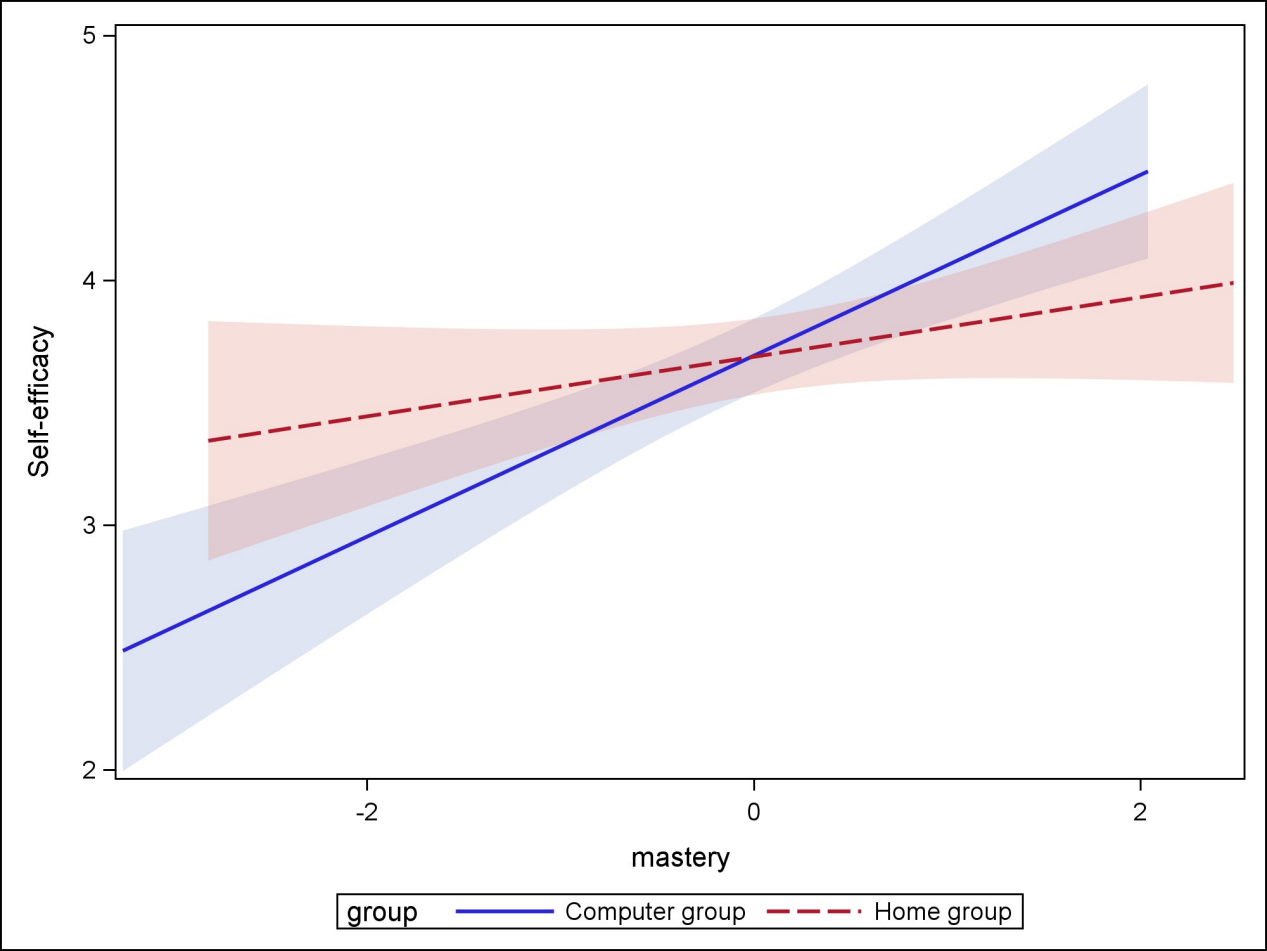
Illustration of the interaction between mastery orientation and self-efficacy for the computer and home groups.

The final three columns of Table 2 indicate that the inclusion of the three goal orientation variables (mastery, performance-approach, and performance-avoid) as co-variates did not lead to differences between the home and classroom groups on the three dependent variables in this study. The effect size difference with mastery orientation as a covariate was 0.11 (95% CI -0.28 to 0.50) for the learning outcome (interaction *p* = 0.9886); and 0.11 (95% CI -0.28 to 0.49) for intrinsic motivation (interaction *p* = 0.3182). However, there was a significant interaction for the outcome of self-efficacy (*p* = 0.0263), where the effect of mastery orientation on self-efficacy was stronger for the computer group (see Fig 1). The effect size difference with performance-approach orientation as a covariate was 0.15 (95% CI -0.24 to 0.54) for the learning outcome (interaction *p* = 0.1424); 0.02 (95% CI -0.39 to 0.43) for intrinsic motivation (interaction *p* = 0.6894); and 0.12 (95% CI -0.28 to 0.53) for self-efficacy (interaction *p* = 0.4265). The effect size difference with performance-avoid orientation as a covariate was 0.16 (95% CI -0.23 to 0.54) for the learning outcome (interaction *p* = 0.4730); 0.00 (95% CI -0.40 to 0.40) for intrinsic motivation (interaction *p* = 0.3229); and 0.11 (95% CI -0.30 to 0.52) for self-efficacy (interaction *p* = 0.6953). This leads us to the conclusion that the lack of differences between the home and the classroom groups on learning outcome, and intrinsic motivation remained after accounting for the three different forms of goal orientation. There was an interaction between mastery orientation and self-efficacy which means that the differences between the groups (computer vs. home) on the outcome of self-efficacy, differed based on mastery orientation. Fig 1 illustrates the interaction between these variables across the groups.

## Discussion

### Empirical contributions

The main empirical contribution of this study is the finding that the equivalence hypothesis—i.e., students learn as well from computer-based interactive science simulations when they are performed at home as when they are performed in class—was supported. Specifically, the students who were assigned the virtual biology laboratory simulation to independently complete at home in an informal environment performed equally well on the learning outcome test as the students who used the simulation in a formal classroom environment with teacher supervision. This result is consistent with the results found in earlier studies [12,13], and add to the evidence that there is equivalence between students who use virtual simulations at home or in a classroom context.

A second empirical contribution was the further demonstration of equivalence for motivation and self-efficacy for the home and classroom groups; i.e., no differences in self-reported motivation or self-efficacy between the students who took the simulation at home and those who took it in a formal classroom. Increasing students’ motivation to learn science has been highlighted as one of the most important potential benefits of using simulations in science education [16]. The results in this study indicate that this need not involve formal supervision by teachers, but can be equally achieved via independent engagement in these simulations. This finding is particularly relevant because there is a growing body of research and interest in the importance of motivation, the social and cultural context, and feelings of self-efficacy in supporting learning in STEM [6,16]. The final major empirical contribution of this paper was the finding that the equivalent outcomes were not dependent on prior knowledge or goal orientation.

### Practical contributions

The main practical contribution of this paper is in developing an evidence-based framework that can help science educators in planning educational activities that optimize learning and motivational outcomes for students. One important area of research on the use of simulations for science learning is the comparison of their value across different educational contexts. This research question is specifically relevant because there is a rapid increase in the use of virtual science lab simulations that are administered online^1^. The results in this study provide initial support to the use of administering unsupervised lab simulations online. Furthermore, in more traditional settings, given the limitations on teacher resources, it is relevant to increase instructional time by assigning online learning activities at home, as homework, blended instruction, or as part of a flipped classroom. The results of this study show that the quality of the outcomes from these activities when they are performed at home is equivalent to the outcomes when they are used in classroom environments, which can free up class time for other relevant activities. Furthermore, the results can have vast ramifications as they suggest that it is possible to make STEM more accessible and inclusive by making effective laboratory activities available to student groups who would not be able participate and have access a physical STEM laboratory.

### Theoretical contributions

The equivalence hypothesis is based on an extension of Clark's [17] distinction between instructional media and instructional methods, in which he emphasizes the importance of the instructional methods impact on learning. In applying the methods-not-media view to the equivalence hypothesis, the instructional method—that is the interactive science simulation—should cause the learning, but the medium or context—home or classroom—in which it is delivered should not. The findings in this study support the equivalence hypothesis because there were no differences in outcomes between the home and classroom contexts.

This is in contrast to more social constructivist theoretical perspectives of learning that consider the context in which the learning occurs as central to learning itself [50]. In the present study, however, context refers simply to the physical location in which a VR activity was undertaken. We conclude that the virtual context in a carefully designed VR science simulation can be so powerful in and of itself that students become immersed in that virtual context and can experience a sense of presence regardless of the environment in which they are physically present. A feeling of presence—which is defined as a psychological state in which virtual (para-authentic or artificial) physical objects, social actors, and the self, respectively, are experienced as actual entities [51]—is, therefore, a very important variable that could mediate the importance of the physical context in which the simulation is being used [52–55]. In this way, the context as experienced by the learner may be equivalent. The findings in this study also support the idea that the principles of multimedia learning [30] that were implemented in the bacterial isolation simulation hold equally well in the two contexts under which they were tested in this study.

### Limitations and future directions

While the results of this study support the equivalence hypothesis and the idea that simulations can be used just as successfully at home, there are some cautions. One limitation was that only 112 of the 289 students enrolled in the biology course (38.75%) agreed to provide their data for the study. Therefore, it is possible that the sample of participating students was biased in some way, such as being the more motivated or mastery oriented students. However, we did demonstrate that differences in prior knowledge or goal orientations, which are known to influence learning outcomes, did not affect the equivalence of the outcomes. We did find that the relationship between mastery orientation and self-efficacy was different between the home and classroom groups. The results suggest that mastery orientation plays a bigger role in predicting self-efficacy when simulations are used in classroom settings compared to at home. Future research should investigate if this finding can be replicated in different samples with different learning material.

There are also several future research efforts needed in order to understand how simulations can be incorporated optimally into the science curriculum. For example, how much and during which activities should teachers invest time in face-to-face activities compared to online learning? How should teachers follow-up on simulation activities in the classroom in terms of exploration, discussions or reflection of the learnt material? How much prior knowledge or preparation do students need before engaging in a simulation in order to get optimal learning and motivational outcomes? Our results showed equivalence across students with different levels of prior-knowledge, but is there a minimal amount of prior knowledge before these are effective learning tools?

Although the results in this study support the equivalence hypothesis, simulations have specifically been shown to be effective for learning when sufficient guidance is given [56]. The Bacteria Isolation simulation used in this study included guidance in the form of concrete instructions by a pedagogical agent, to ensure that students progressed smoothly through the simulation activities. We reason that when a virtual context is developed in a way to achieve enough psychological presence, students will perceive the virtual environment to be their primary learning context. When this happens, optimizing contextual factors within the virtual environment becomes important for learning [42] and the physical environment becomes less important. However, more research is needed to investigate likely boundary conditions to the equivalence hypothesis, as there are potentially settings where individualized classroom guidance is more effective than the guidance that is currently possible in a virtual environment. Future research should also investigate the role of this guidance and how to individualize it so that it is optimized to each student’s learning and motivational needs.
